# Disaster-related information support systems for families of older adults with special healthcare needs: A scoping review

**DOI:** 10.1371/journal.pone.0358320

**Published:** 2026-10-16

**Authors:** Qianmei Zhong, Chunyue Xiao, Dan Liu, Lawrence T. Lam, Jia Pu

**Affiliations:** 1 Department of Immunology, Mianyang Central Hospital, Affiliated with the School of Medicine, University of Electronic Science and Technology of China, Mianyang, China; 2 School of Public Health, Macau University of Science and Technology, Macau, China; STIKES Wira Medika PPNI Bali: Sekolah Tinggi Ilmu Kesehatan Wira Medika PPNI Bali, INDONESIA

## Abstract

Based on the theory of structural vulnerability, this study systematically examines the characteristics and underlying mechanisms shaping information needs among families of older adults with special healthcare needs in disaster contexts, providing a theoretical foundation for developing a family-system collaborative disaster information support system. In this scoping review, we systematically searched PubMed, Embase, Web of Science, Cochrane Library, China National Knowledge Infrastructure (CNKI), Wanfang Data, VIP Database, and the Chinese Biomedical Literature Database from inception to December 31, 2025. A total of 22 studies were included. The findings suggest that the information needs in this population are highly heterogeneous, particularly among families managing cognitive impairments and chronic diseases. These families not only bear the responsibility of information transmission but also act as intermediaries responsible for translating, interpreting, and filtering information. However, existing disaster information support systems insufficiently meet their needs, particularly regarding information accessibility and comprehensibility, which presents significant gaps. Additionally, family caregivers face a disparity between responsibility and capacity, lacking effective support tools, potentially contributing to decision fatigue and information overload. Overall, the study suggests that the information needs of these families may arise from structural mechanisms embedded within disaster response systems. Future disaster information support strategies should move beyond the simple “family-centered” approach to a family-system synergy model. Such an approach should incorporate decision-support toolkits and parallel communication channels while also providing structured decision support and risk-sharing mechanisms. This shift would transform family caregivers from unsupported gap-fillers into active yet supported decision participants, thereby enhancing both disaster response capacity and equity for this vulnerable population.

## Introduction

As the frequency of global disasters increases alongside population aging, ensuring that older adults can timely and accurately access early warning information, emergency measures, and medical resources has become an essential task within disaster management systems [1,2]. According to the World Health Organization, individuals aged 65 and older account for over 50% of disaster-related fatalities [3]. Among them, older adults with special healthcare needs, including those who depend on long-term care and suffer from chronic conditions or cognitive impairments, are particularly vulnerable due to functional decline, medication dependence, and limited social support [4]. However, existing disaster management and emergency response systems tend to focus more on macro-level resource allocation, with limited attention given to the unique information acquisition and understanding needs of this group [5]. In disaster contexts, the family serves as the primary support and decision-making unit, where family caregivers not only handle daily care responsibilities but also engage in information filtering, risk assessment, and decision-making [6,7]. Therefore, there is a need to shift the analytical focus from the individual to the family level, and to systematically examine information needs and support mechanisms for families in disaster settings. Based on this rationale, the present study applies the theory of structural vulnerability [8] and scoping review methodology [9] to systematically analyze the information needs among families of older adults with special healthcare needs, thereby providing a theoretical foundation for developing structured disaster information support system.

## Materials and methods

This scoping review was conducted in accordance with the Arksey and O’Malley [9] methodological framework and reported following the Preferred Reporting Items for Systematic Reviews and Meta-Analyses extension for Scoping Reviews (PRISMA-ScR) checklist (S1-S2 File). This research is exempt from Research Ethics Board review as it involves analysis of previously published literature.

### Theoretical framework

This study applied the theory of structural vulnerability to guide data extraction, evidence synthesis, and interpretation of findings. This theory posits that health-related vulnerability is not an inherent individual attribute, but is constructed through power relations arising from the interaction between social structures and individuals [8]. Specifically, the theory informed this review in three ways. First, it shaped the definition of disaster information support system as a structured socio-technical platform that integrates multiple channels—including digital tools, community support, and medical service networks—to provide timely, accurate, and adaptive disaster information to families of older adults with special healthcare needs. Second, it directed analytical attention to the structural mechanisms—rather than individual-level factors—that produce information disparities, such as the asymmetry between caregiver responsibility and capacity, non-voluntary functional transfer of information labor from formal systems to families, and digital divide as a manifestation of structural inequality. Third, it provided a conceptual lens for interpreting how power relations embedded within disaster response systems shape families’ information access, processing, and decision-making. The system performs key functions, including information filtering, translation, coordination, and decision support, thereby bridging the structural gap between formal systems and families [10].

### Analysis method

This study adopted a theoretically informed inductive approach for its analysis strategy, structured in three sequential stages. In the first stage (open coding), two researchers (QZ and PJ) independently coded the included studies using a standardized extraction form, collecting data on information needs, information sources, key barriers, and main findings. In the second stage (thematic synthesis), the coded data were grouped into thematic categories through iterative comparison, identifying recurring patterns across studies. In the third stage (theory-informed interpretation), the inductively derived themes were examined through the lens of structural vulnerability theory to identify the underlying structural mechanisms—such as non-voluntary functional transfer, responsibility—capacity asymmetry, and structural information exclusion—that shape families’ information needs and access patterns. This approach ensured that the analysis moved beyond descriptive categorization toward theoretically grounded interpretation of the structural determinants of information vulnerability in disaster contexts.

### Information sources & search tools

A comprehensive literature search was conducted across the following databases: PubMed, Embase, Web of Science, the Cochrane Library, China National Knowledge Infrastructure (CNKI), Wanfang Data, VIP Database, and the Chinese Biomedical Literature Database (CBM). The search covered the period from database inception to December 31, 2025. The search was performed by combining subject headings (MeSH terms) with free-text terms across the databases. The complete search strategies for PubMed and other databases are provided in S1 Table and S3 Table.

### Eligibility criteria

Based on the PCC framework (Participants, Concept, Context) [11], the inclusion criteria for this review were as follows:

(1) Participants: older adults aged ≥60 years with special healthcare needs and their families, such as those with chronic diseases, multimorbidity, functional impairments, cognitive disorders, and disabilities or those requiring long-term medical care;
(2) Concept: original studies addressing disaster-related information needs, information-seeking behaviors, risk communication, or early warning information under disaster conditions;
(3) Context: disaster settings, including natural disasters or public health emergencies, such as earthquakes, floods, typhoons, extreme heat events, and infectious disease outbreaks.

The exclusion criteria were as follows:

(1) duplicate publications;
(2) studies for which the full text were unavailable;
(3) studies published in other languages apart from Chinese or English.

### Study selection and data extraction

Duplicate references were removed using NoteExpress software. Two trained researchers (QZ and JP) independently conducted literature screening and data extraction according to the inclusion and exclusion criteria. In the initial screening phase, titles and abstracts were reviewed. In the second phase, full-text articles were assessed for eligibility. In case of disagreement, consensus was reached through discussion, and a third researcher (DL) was consulted when necessary. During the data extraction and analysis process, the research team developed a standardized data extraction form, which included study basic information, characteristics of the study population, disaster types, information needs, information sources, key barriers, and intervention measures, among others. To ensure consistency and rigor in the analysis, the following measures were adopted: 1) dual independent coding and cross-checking by two trained researchers (QZ and LL), with disagreements resolved through discussion and consultation with a third researcher (CY), to improve result consistency; 2) continuous comparison of themes and concepts across studies during the analysis process to form a stable thematic framework; and 3) retention of complete data extraction and analysis records to enhance transparency and traceability of the research process.

## Results

### Literature search results

After removing duplicate records, 2,913 articles remained. After title and abstract screening, 53 articles were retained for full-text review. Ultimately, 22 studies met the inclusion criteria and were included in this review [12–33]. The study selection process is illustrated in S1 Fig.

### Basic characteristics of the included studies

A total of 22 studies were included in this research [12–33], comprising 11 cross-sectional studies [15,18,20,21,24,27–30,32,33], 7 qualitative studies [12–14,16,17,23,25], 2 randomized controlled trials [22,26], and 2 longitudinal studies [19,31]. The study participants mainly consisted of older adults with special healthcare needs and their family caregivers, covering individuals with cognitive impairments, chronic diseases, and mental health. The research primarily focused on the information needs in disaster or public health emergency situations, including information access channels, the process of information comprehension and translation, communication barriers, and information support strategies. The basic characteristics of the included studies are presented in S2 Table.

### Thematic analysis of disaster information needs among families of older adults with special healthcare needs

#### Disaster risk and early warning information needs.

Across disaster contexts, families of older adults with special healthcare needs suggest a strong reliance on timely, clear, and actionable risk and early warning information. Evidence suggests that during the pre-disaster and early response phases, family caregivers particularly require concise and comprehensible information to support rapid decision-making. Heppenstall et al. [23] reported that unclear information or communication barriers can lead to heightened anxiety and uncertainty among both older adults and caregivers. For individuals with cognitive or language impairments, family members often need to simplify and reinterpret official information [13,14,16]. This “double translation” burden may reflect a lack of cognitively accessible communication design within information systems, effectively shifting the responsibility for information adaptation onto families.

#### Medical continuity and health management information needs.

Studies suggest that families prioritize information related to continuity of care, medication supply, and alternative access to healthcare services during disasters [15,17,21,25,28,33]. Kyota et al. [21] reported that families of older adults on long-term medication express strong needs for emergency drug stockpiling and post-disaster access pathways, reflecting limited trust in the responsiveness of healthcare systems. This pattern of “redundant information seeking” may indicate that families actively compensate for systemic shortcomings in medical logistics. Although telemedicine and contactless prescription services serve as important supplements, reliance on these informal or alternative channels also exposes gaps in formal healthcare continuity planning, thereby placing additional management and decision-making burdens on families.

#### Care and support service information needs.

During disasters, family caregivers suggest increased demand for information on community support, social services, and formal care resources [13,17,18,23,33]. In emergency contexts such as pandemics, needs related to medication delivery, essential supplies, and home-based care support become particularly prominent [17,18,33]. This rise in demand may reflect a shift of coordination responsibilities from public systems to families. As a result, caregivers are compelled to undertake “service coordination labor” that would otherwise be managed at the system level, thereby increasing their cognitive and decision-making burden.

#### Information access channels and communication needs.

Families access disaster-related information through multiple channels, including traditional media (television, radio, telephone) and information and communication technology (ICT)-based platforms (telemedicine, social media, e-government services) [12,18,24,29,31]. DeJohn et al. [12,24] found that although ICT use increased significantly during the pandemic, traditional media remained more trusted among older adults, particularly in families with cognitive impairment or low digital literacy. Caregivers frequently act as intermediaries in accessing and interpreting information [13,16,19,32]. However, this “mediated information access model” may represent a non-voluntary functional shift. Due to the lack of direct communication pathways to cognitively or linguistically vulnerable older adults, families must assume both information filtering and decision-making responsibilities, while also bearing the risks associated with potential misinterpretation.

#### Psychological support and social connectivity information needs.

Evidence suggests that disasters significantly increase the need for information related to mental health support and social connectivity among older adults and their families [12,14,22,26,30]. Vives-Rodriguez et al. [22,26] suggested that remote communication can help alleviate loneliness and psychological distress, particularly during periods of isolation. As access to professional mental health services declines, families may increasingly need to assume responsibility for emotional support, further extending their caregiving role.

### Disaster information support strategies and interventions

#### ICT-based support strategies.

ICT-based strategies play a critical role during disasters, including telemedicine, video communication, online health education, e-prescriptions, and medication delivery services [12,15,22,25,28,31,32]. These interventions contribute to maintaining continuity of care, providing health guidance, and facilitating social connectivity. Evidence suggests that appropriate use of remote communication technologies can enhance information accessibility and support families in coping with disaster-related uncertainties [22,26,31]. However, such strategies are often built on the assumption of “resource-sufficient households,” overlooking the needs of families affected by cognitive impairment or limited digital access, thereby potentially exacerbating health inequities.

#### Information literacy and capacity-building interventions.

Improving information literacy and technological competence is an important approach to strengthening families’ disaster response capacity [20,29]. Through health education programs and technical training, older adults and their caregivers can enhance their ability to access, evaluate, and apply information. Nevertheless, this “capacity-oriented” approach may overlook structural communication barriers, tending to attribute communication difficulties to individual limitations rather than deficiencies in system design. Consequently, the structural barriers underlying these difficulties remain unaddressed.

#### Caregiver-oriented information support strategies.

Several studies emphasize caregivers as primary targets of disaster information support, recommending structured information resources, standardized care pathways, and simplified emergency procedures to reduce the need for information filtering and alleviate decision-making burdens [16,17,25]. Some studies also emphasize caregivers’ needs for emotional support and peer networks in the communication process [17,26]. However, the intermediary work performed by caregivers is often overlooked. Information processing involves both functional tasks and substantial cognitive and emotional labor. Policy recommendations that encourage families to “stay informed” may neglect the fact that information overload itself has become a major stressor [17,30]. More importantly, the risks associated with information mediation are largely borne by families. This asymmetry between responsibility and capacity may constitute a core mechanism underlying the disaster vulnerability of families with special healthcare needs.

#### Community- and system-level information support measures.

Evidence suggests that integrating healthcare, social services, and public health resources into unified information platforms can improve the coherence and accessibility of information delivery [18,23,27,33]. However, current institutional arrangements still rely heavily on informal family support, increasing the burden on families and limiting the system’s intended supportive function. Heppenstall et al. [23] found that when formal emergency systems fail to directly reach older adults with cognitive impairments, families become the sole communication interface. This dependency may exhibit a self-reinforcing pattern: the more the system relies on family intermediaries, the less incentive there is to improve direct communication channels; and the more families compensate for system gaps, the greater their burden becomes.

## Discussion

### Complexity of information filtering and translation

This study found that families of older adults with special healthcare needs appear to have a strong demand for timely, clear, and actionable disaster risk and early warning information, particularly during preparedness and early response phases. Family caregivers rely heavily on concise and practical information to make urgent decisions. However, the findings also reveal structural deficiencies in disaster information systems: when information is unclear or communication is disrupted, both older adults and caregivers experience heightened anxiety and uncertainty, while frequent updates and inconsistent policies further increase the burden of information processing. This challenge is especially pronounced among older adults with cognitive or language impairments, where caregivers must simplify and reinterpret official information. The resulting “double translation” burden, along with the “triple translation” dilemma faced by linguistic minority groups—across language, culture, and cognitive barriers—may substantially increases caregivers’ cognitive and communicative workload [34]. This phenomenon aligns with clinical communication theory, which highlights how information distortion and processing barriers hinder older adults’ ability to accurately and promptly understand disaster-related information [35]. Furthermore, age-related cognitive decline and low health literacy further impair information decoding, thereby increasing caregivers’ cognitive burden and emotional stress during disaster response [36]. These findings suggest that disaster information support systems should prioritize the needs of vulnerable groups, particularly those with cognitive impairments, by adopting universal design principles to create more accessible and inclusive communication strategies. Such approaches can reduce the non-voluntary translation burden on caregivers and reposition them from passive gap-fillers to supported participants in decision-making.

### High demand for medical continuity and health management information

This study suggests that families of older adults with special healthcare needs have particularly strong demands for access to healthcare services, medication supply, and alternative care pathways during disasters. In many cases, proactive efforts by families compensate for gaps in medication security and continuity of care within formal systems. The high demand for drug stockpiling and alternative access routes may reflect a lack of trust in existing healthcare systems, especially regarding medical logistics and emergency responsiveness. This pattern of “redundant information seeking” suggests that when formal emergency systems fail to provide adequate support, families are compelled to act as primary agents of self-management, translating systemic insufficiencies into preventive labor at the household level [37]. Although informal channels such as telemedicine and contactless prescription services can provide partial support, overreliance on these approaches may introduce new challenges, particularly concerning the accuracy and reliability of information [38]. The structural paradox identified by Olszewski et al. [29] further highlights that those with the greatest need for continuity of care, such as individuals with low education levels, advanced age, or cognitive impairments, are often excluded from digital access channels. These findings highlight the need for disaster preparedness systems to prioritize the continuity of healthcare services by strengthening emergency medical logistics and ensuring stable access to medications. Reducing dependence on informal channels is particularly critical for low-resource households with limited access to digital health services. Establishing centralized information coordination mechanisms that integrate fragmented healthcare and social service information may help ensure the accessibility and reliability of critical information during disasters.

### Asymmetry between caregiver responsibility and capacity

Disaster preparedness theory emphasizes the critical role of families, particularly during the early stages of disaster response [39]. However, this review reveals that families are not merely passive recipients of information; they undertake substantial, often unquantified cognitive labor, including real-time risk translation, cultural adaptation, emotional support, and the intermediary work of information filtering, interpretation, and coordination. These efforts are rarely accounted for in the formal costs of disaster response, yet they consume significant physical and psychological resources of caregivers. More importantly, there exists a pronounced asymmetry between responsibility and capacity. While families bear ultimate decision-making responsibility, they often lack adequate support tools and decision aids. Under conditions of information overload and uncertainty, this imbalance may contribute to decision fatigue [40, 41]. Evidence suggests that when formal emergency systems fail to directly reach vulnerable populations, families are compelled to assume coordination and decision-making roles that should be fulfilled by the system, thereby compensating for structural gaps. This unplanned functional shift may intensify caregivers’ cognitive and emotional burden, and may also result in the neglect of critical healthcare needs of vulnerable older adults, creating a structural dilemma of “acknowledged but unsupported” care [42]. Addressing this issue requires strengthening community-based support systems and improving the integration of social and healthcare services. Establishing unified information platforms and coordinated service pathways can enhance accessibility and reduce fragmentation. Future disaster information support systems should prioritize system–community coordination, providing centralized and structured information resources, clearly defined care pathways, and accessible support services. Such efforts can alleviate caregivers’ burden and transform families from unsupported intermediaries into supported decision partners.

### Increased demand for psychological support–related information

With the growing need for mental health support and social connectivity during disasters, family caregivers are increasingly compelled to assume emotional support roles, particularly when access to professional mental health services is limited. Evidence suggests that maintaining social interaction through remote communication and virtual platforms can help alleviate loneliness and psychological distress [43]. However, the emotional labor involved in providing such support, which is traditionally the responsibility of professional services, is now being shifted onto family caregivers, representing an unplanned shift of professional responsibilities onto families, representing an unplanned shift of professional responsibilities onto families. These findings indicate that disaster information support systems should explicitly incorporate mental health support components, including integrating accessible psychological resources such as tele-mental health services and virtual social support platforms. In addition, governments and social service agencies should provide targeted training and professional emotional support for caregivers, enabling them to better manage psychological burdens while reducing the risk of stress transmission and the amplification of mental health problems within families.

### Dual-track differentiation in information access channels

This study identifies a “dual-track differentiation” pattern in disaster information access among families of older adults with special healthcare needs. While ICT-based platforms (e.g., telemedicine, video communication, e-government services) saw increased use during the pandemic, traditional media (e.g., television, radio, telephone) remain dominant among populations with cognitive impairments or low digital literacy due to their simplicity and familiarity. In this context, family caregivers play an intermediary role, maintaining contact with healthcare providers and community organizations, often via telephone, to obtain updated information and facilitate its interpretation and communication within the household. However, this “mediated information access model” represents a non-voluntary functional shift [44]. In the absence of direct communication channels tailored to cognitively or linguistically vulnerable older adults, families often become de facto information gatekeepers, assuming additional responsibilities in information translation, risk assessment, and service coordination [34]. These findings suggest that disaster response systems should prioritize the diversification and adaptability of communication channels by developing resilient communication networks and expanding direct access points, such as professional hotlines and accessible telehealth services, particularly for cognitively impaired and low–digital literacy groups. At the same time, systems should reduce reliance on family intermediaries by providing simplified information formats (e.g., pre-filtered information packages, visual aids) and more direct communication pathways. Such measures can reduce caregiver burden and enable families to participate in decision-making with adequate structural support rather than as de facto information brokers.

### From family-centered to family–system synergy

This study proposes a shift from a “family-centered” model to a “family–system synergy” approach, conceptualizing disaster information support systems as structured mechanisms composed of information provision, communication pathways, and decision support. At the system level, disaster information support should move beyond one-way information delivery toward proactive support [45]. On the one hand, multimodal information design can reduce the burden of “double translation” and improve accessibility for individuals with cognitive or language impairments. On the other hand, establishing parallel communication mechanisms (e.g., professional hotlines, community-based interfaces) can reduce overreliance on family intermediaries. In addition, systems should incorporate risk-sharing and error-correction mechanisms by providing pre-filtered, standardized information resources and clarifying institutional responsibility for information quality, thereby reducing uncertainty in family decision-making processes. At the family level, support components should be embedded within the system to enhance caregivers’ information-processing capacity. This includes providing pre-screened and pre-interpreted information packages, as well as offering community-based support or technical assistance to bridge the digital divide. Through such system-oriented interventions, families can shift from unaided information brokers to supported collaborators in the decision-making process, ultimately improving information accessibility and strengthening overall disaster response capacity.

### COVID-19 specificity and boundaries of external validity

This review is primarily grounded in the COVID-19 context; therefore, caution is warranted when generalizing these findings to natural disasters such as earthquakes and floods. Unlike acute disasters, COVID-19 is characterized by a prolonged and cumulative trajectory, during which families experience sustained “redundant information seeking.” In contrast, natural disasters are typically sudden and short-term events, where information needs are concentrated on immediate warning and evacuation decisions. Despite these contextual differences, both scenarios may share a structurally similar mechanism of “non-voluntary functional transfer,” whereby families are required to fill gaps in formal communication and support systems. Another key distinction lies in infrastructure dependence. During the COVID-19 pandemic, relatively stable infrastructure enabled ICT-based platforms to become the dominant information channels, thereby amplifying “digital dual-track differentiation.” In contrast, natural disasters may disrupt critical infrastructure, thereby weakening digital channels and forcing families to rely more heavily on traditional media and neighborhood networks. Future research should examine the “family–system synergy” model under conditions of infrastructure disruption and explore how multi-channel integration and optimized information delivery can mitigate the impacts of digital exclusion and information overload on families.

## Limitations of the study

This study has several limitations that should be considered when interpreting the findings. First, there is a contextual evidence bias, as 86.4% of the included studies focus on the COVID-19 pandemic. This may overrepresent an “infrastructure-intact” chronic crisis setting while underrepresenting acute disasters such as earthquakes and floods, where electricity and communication networks are often disrupted. Second, most included studies employed cross-sectional designs, limiting the ability to capture longitudinal changes in household information-processing labor over time. Third, only English- and Chinese-language publications were included, which may have led to the omission of relevant evidence from other linguistic and cultural contexts regarding family information practices. Fourth, in accordance with scoping review methodology, formal quality appraisal of individual studies was not conducted; consequently, studies of varying methodological rigor were included, and the strength of evidence underlying each theme may differ. Fifth, the heterogeneity of study designs, populations, and outcome measures across the included studies precluded direct comparative analysis and may limit the precision of the synthesized themes. These limitations constrain the generalizability of the findings to non-pandemic disaster settings, particularly the applicability of conclusions related to ICT dependence under conditions of infrastructure disruption.

## Conclusions

This review suggests the information needs of families of older adults with special healthcare needs in disaster contexts and explores possible theoretical mechanisms through the lenses of clinical communication, disaster preparedness, and caregiving theories. The proposed disaster information support system aims to provide structured disaster information services and improve information accessibility and service feasibility through multi-channel access, decision-support functions, and cross-sectoral coordination mechanisms. Future efforts should focus on developing a context-adaptive disaster information support system and promoting a shift from a “family-centered” approach toward a “family–system synergy” model. Through structured decision support and risk-sharing mechanisms, families can transition from unsupported intermediaries to adequately supported decision partners. Future research should further validate this theoretical framework in non-pandemic disaster contexts, particularly by examining patterns of channel differentiation under conditions of infrastructure disruption.

## Supporting information

S1 Fig Flow Chart for Literature Search and Screening. (DOCX)

S1 Table Search strategy for the study. (DOCX)

S2 Table Basic Characteristics of the Included Studies (n = 22). (DOCX)

S3 Table Retrieval strategies for other databases. (DOCX)

S1 File Human Participants Research Checklist. (DOCX)

S2 File PRISMA-ScR Checklist. (DOCX)
