# Supplementary material for: Disaster-related information support systems for families of older adults with special healthcare needs: A scoping review
Source: PLoS One. 2026 Oct 16;21(10):e0358320. doi: 10.1371/journal.pone.0358320 (PMC13674750; doi:10.1371/journal.pone.0358320)
Supplement: Supplementary file 1 [file pone.0358320.s001.docx]

**S1 Figure.**

Records identified through database searching (n=3864):

CBM (n=9), PubMed (n=508), Embase (n=2611), Cochrane library (n=86), Web of Science (n=603), EBSCO (n = 25), CNKI (n=7), Wangfang (n=15)

Records after duplicates removed (*n*=951)

Read the title and abstract for preliminary screening (*n*=2913)

Read the full rescreen (n=53)

））

Included literature for analysis (n=22)


Records excluded (n=2860):

Not published in Chinese or English (N=5);

Subject irrelevance (n=1794);

Reviews, conference abstracts, or other non-original research (n=485)

Incomplete abstract information (n=576)

Records excluded (n=31):

Irrelevant study content (n=29);

Full text not available (n=2)

Fig 1. Flow Chart for Literature Search and Screening.
