# Supplementary material for: Disaster-related information support systems for families of older adults with special healthcare needs: A scoping review
Source: PLoS One. 2026 Oct 16;21(10):e0358320. doi: 10.1371/journal.pone.0358320 (PMC13674750; doi:10.1371/journal.pone.0358320)
Supplement: Supplementary file 2 [file pone.0358320.s002.docx]

**S1 Table**

**Table 1. Search strategy for the study**

| Item | Content | | |
| --- | --- | --- | --- |
| Data sources | PubMed | | |
| Search strategy | #1 | 3,980,650 | ("Aged"[Mesh] OR "older adult*" OR "elderly" OR "aging population" OR "senior" ) |
|  | #2 | 647,065 | ("Special Healthcare Need*" OR "medically complex" OR "chronic disease" OR "disability" OR "long-term care" OR "home care" OR "family caregiver*" OR "chronic disease*" OR "disab*" OR "cognitive impairment" OR "medical depend*" OR "caregiver") |
|  | #3 | 1,161,240 | ("Disaster*"[Mesh] OR "emergency" OR "hazard*" OR "flood" OR "earthquake" OR "pandemic") |
|  | #4 | 433,806 | ("information need*" OR "risk communication" OR "warning information" OR "emergency communication" OR "communication" OR "health information" OR "information behavior" OR "information seeking" OR "information access" OR "health communication") |
|  | #5 | 508 | #1 AND #2 AND #3 AND #4 |
