# Supplementary material for: Disaster-related information support systems for families of older adults with special healthcare needs: A scoping review
Source: PLoS One. 2026 Oct 16;21(10):e0358320. doi: 10.1371/journal.pone.0358320 (PMC13674750; doi:10.1371/journal.pone.0358320)
Supplement: Supplementary file 3 [file pone.0358320.s003.docx]

**S2 Table**

**Table 2. Basic Characteristics of the Included Studies (n = 22)**

| **Included literatures** | **Country** | **Study Design** | **Sample Characteristics** | **Disaster Type** | **Key Information Needs** | **Information Sources/Channels** | **Key Barriers** | **Main Findings** |
| --- | --- | --- | --- | --- | --- | --- | --- | --- |
| DeJohn et al. [12] (2024) | United States | Qualitative | Older adults with loneliness and health measures (n=24) | COVID-19 | Information access channels; ICT use patterns | ICT; Traditional media | Low digital literacy; cognitive impairment | ICT use increased during pandemic, but traditional media remained more trusted, especially among those with cognitive impairment or low digital literacy |
| Caprioli et al. [13] (2025) | United Kingdom | Qualitative | Social care providers (n=14), older adults with dementia (n=6), family carers (n=2) | COVID-19 | Information simplification and reinterpretation; dementia-related support services | ICT | Communication barriers for cognitive/language impairments; double translation burden on families | Family members need to simplify and reinterpret official information for individuals with cognitive impairments; ICT used for dementia support services |
| Barnard et al. [14] (2025) | New Zealand | Qualitative | Isolated older adults with cognitive impairment (n=24) | COVID-19 | Mental health support; social connectivity | Community support networks | Language and literacy barriers; social isolation | Disasters increase need for mental health support and social connectivity; language and literacy barriers compound information access challenges |
| Tan et al. [15] (2022) | Malaysia | Cross-sectional | Older adults (n=42) and caregivers (n=197) | COVID-19 | Continuity of care; medication supply; alternative healthcare access | Virtual medical consultations | Healthcare service disruption; access barriers | Families prioritize information on continuity of care, medication supply, and alternative healthcare access during disasters |
| Shafer et al. [16] (2023) | United States | Qualitative | Spouses (n=7), family members (n=5), close friends (n=1) of stroke survivors with aphasia | COVID-19 | Information access; role adaptation for care partners | Multiple information channels | Aphasia-related communication barriers; role transition challenges | Caregivers act as intermediaries in accessing and interpreting information; aphasia creates unique communication challenges for information access |
| Sadler et al. [17] (2020) | United States | Qualitative | Family caregivers of older adults with cognitive impairments (n=14) | COVID-19 | Continuity of care; community support; medication delivery; essential supplies | Remote healthcare tools | Healthcare service disruption; caregiver burden; cognitive impairment | Families show increased demand for information on community support, formal care resources, medication delivery, and home-based care support |
| Golubeva et al. [18] (2022) | Russia | Cross-sectional | Caregivers of older people (n=90) | COVID-19 | Community support; medication delivery; essential supplies; self-isolation management | ICT; Traditional media | Self-isolation challenges; healthcare access barriers | Families access information through multiple channels; caregivers show increased demand for community support and service coordination during self-isolation |
| Giles et al. [19] (2025) | United States | Longitudinal | Older adults with dementia (n=186) | COVID-19 | Information access; communication support | Virtual reality interface | Data collection challenges for patients with dementia; cognitive impairment | Caregivers act as intermediaries in accessing and interpreting information; methodological challenges in studying older adults with dementia during disasters |
| Li et al. [20] (2022) | China | Cross-sectional | Older adults with dementia (n=617) | COVID-19 | Health information literacy | Multiple information sources | Low health information literacy; cognitive impairment | Health information literacy is a significant concern among community-dwelling older adults during the pandemic |
| Kyota et al. [21] (2021) | Japan | Cross-sectional | Family caregivers of older adults on oral medications (n=58) | Natural disasters | Emergency drug stockpiling; post-disaster medication access; disaster preparedness | Multiple information sources | Limited trust in healthcare system responsiveness; disaster preparedness gaps | Families of older adults on long-term medication express strong needs for emergency drug stockpiling and post-disaster access pathways |
| Vives-Rodriguez et al. [22] (2024) | United States | Randomized controlled study | Cognitively unimpaired (n=77) or cognitively impaired (n=85) older adults | COVID-19 | Mental health support; social connectivity; remote communication | Remote communication technologies | Cognitive impairment; social isolation | Remote communication can help alleviate loneliness and psychological distress; ICT strategies enhance information accessibility |
| Heppenstall et al. [23] (2013) | New Zealand | Qualitative | Older adults (n=50), caregivers (n=34) | Earthquake | Mental health; communication; evacuation information | Multiple information sources | Unclear information; communication barriers; evacuation challenges | Unclear information or communication barriers lead to heightened anxiety and uncertainty among older adults and caregivers during earthquakes |
| Park et al. [24] (2023) | South Korea | Cross-sectional | Older adults with mental health issues (n=9301) | COVID-19 | Information access channels; ICT use; mental health support | ICT; Traditional media | Digital divide; mental health challenges | ICT use increased during pandemic but traditional media remained more trusted; ICT accessibility affects mental health outcomes for older adults |
| Klinton et al. [25] (2023) | Canada | Qualitative | Caregivers of older adults with dementia (n=4) | All hazards | Continuity of care; telemedicine access; dementia care support | Telemedicine | Telemedicine adoption barriers; dementia care challenges | Telemedicine serves as important supplementary channel for information access; families prioritize information on continuity of care for dementia patients |
| Yu et al. [26] (2021) | United States | Randomized controlled clinical trial | Older adults with mild cognitive impairment (n=100) and normal cognition (n=86) | COVID-19 | Social isolation prevention; cognitive decline mitigation | Internet-based conversational engagement | Social isolation; cognitive decline; technology access barriers | Remote communication technologies help alleviate loneliness and enhance information accessibility; ICT-based interventions support cognitive health |
| Hogeveen et al. [27] (2023) | Canada | Cross-sectional | Older adults with disabilities (n=942) | COVID-19 | Health vulnerability screening; access to care information | interRAI COVID-19 Vulnerability Screener | Disability-related access barriers; vulnerability assessment needs | Health surveillance tools identify vulnerable older adults; disability creates unique information and access challenges during disasters |
| Cheng et al. [28] (2023) | China | Cross-sectional | Older adults with mental health issues (n=110) | COVID-19 | Mental health support; telemedicine access; continuity of care | Telemedicine (SMART system) | Mental health service disruption; technology access barriers | Telemedicine-based strategies support continuity of care; families prioritize information on continuity of mental healthcare services |
| Olszewski et al. [29] (2023) | Poland | Cross-sectional | Older adults with cardiovascular diseases (n=303) | COVID-19 | Sources of COVID-19 information; e-health competencies | Traditional media; ICT; Chatbot | Low education levels; advanced age; cognitive impairments; digital exclusion | Those with greatest need (low education, advanced age, cognitive impairments) are often excluded from digital access channels; structural paradox in information access |
| Ahmmad et al. [30] (2023) | United States | Cross-sectional | Older adults with depression (n=3181) | COVID-19 | Social engagement; health protocol information; social interaction | Social engagement activities; online platforms | Depression; social isolation; digital literacy barriers | Social engagement activities and health protocol maintenance affect depression levels; need for mental health support information during disasters |
| Mao et al. [31] (2023) | United Kingdom | Longitudinal | Older adults (n=2607) | COVID-19 | ICT and telemedicine access; health information | ICT; Telemedicine | Digital divide; technology adoption barriers | ICT use and telemedicine adoption increased during the pandemic; families access information through multiple channels; digital divide persists |
| Yoon et al. [32] (2023) | United States | Cross-sectional | Older adults with dementia (n=153) | COVID-19 | Video call usage; social connectivity; communication support | Video communication technology | Dementia-related technology barriers; digital literacy | Video call usage among older adults with dementia; caregivers act as intermediaries; ICT strategies support information access and social connectivity |
| Zhong et al. [33] (2023) | China | Cross-sectional | Older adults with chronic diseases (n=360) | COVID-19 | Home care demand; continuity of care; community support; medication access | Multiple information sources | Chronic disease management; healthcare access barriers; information fragmentation | Families prioritize information on continuity of care, community support, medication delivery, and home-based care; increased demand for service coordination information |

ICT, information and communication technology; COVID-19, coronavirus disease 2019; interRAI CVS, COVID-19 Vulnerability Screener
