# Supplementary material for: Disaster-related information support systems for families of older adults with special healthcare needs: A scoping review
Source: PLoS One. 2026 Oct 16;21(10):e0358320. doi: 10.1371/journal.pone.0358320 (PMC13674750; doi:10.1371/journal.pone.0358320)
Supplement: Supplementary file 4 [file pone.0358320.s004.docx]

**S3 Table**

**Retrieval strategies for other databases**

| Item | Content | | |
| --- | --- | --- | --- |
| Data sources | WOS | | |
| Search strategy | #1 | 1,579,772 | TS=("Aged" OR "older adult*" OR "elderly" OR "aging population" OR "senior" ) |
|  | #2 | 883,681 | TS=("Special Health Care Need*" OR "medically complex" OR "chronic disease" OR "disability" OR "long-term care" OR "home care" OR "family caregiver*" OR "chronic disease*" OR "disab*" OR "cognitive impairment" OR "medical depend*" OR "caregiver") |
|  | #3 | 1,851,879 | TS=("Disaster*" OR "emergency" OR "hazard*" OR "flood" OR "earthquake" OR "pandemic") |
|  | #4 | 1,516,535 | TS=("information need*" OR "risk communication" OR "warning information" OR "emergency communication" OR "communication" OR "health information" OR "information behavior" OR "information seeking" OR "information access" OR "health communication") |
|  | #5 | 603 | #1 AND #2 AND #3 AND #4 |

| Item | Content | | |
| --- | --- | --- | --- |
| Data sources | Embase | | |
| Search strategy | #1 | 7,215,170 | 'aged'/exp OR 'aged' OR 'older adult*' OR 'elderly'/exp OR 'elderly' OR 'aging population' OR 'senior' |
|  | #2 | 1,576,228 | 'special health care need*' OR 'medically complex' OR 'chronic disease' OR 'disability' OR 'long-term care' OR 'home care' OR 'family caregiver*' OR 'chronic disease*' OR 'disab*' OR 'cognitive impairment' OR 'medical depend*' OR 'caregiver' |
|  | #3 | 2,208,072 | 'disaster'/exp OR 'emergency' OR 'hazard*' OR 'flood' OR 'earthquake' OR 'pandemic' |
|  | #4 | 948,490 | 'information need*' OR 'risk communication' OR 'warning information' OR 'emergency communication' OR 'communication' OR 'health information' OR 'information behavior' OR 'information seeking' OR 'information access' OR 'health communication' |
|  | #5 | 2611 | #1 AND #2 AND #3 AND #4 |

| Item | Content | | |
| --- | --- | --- | --- |
| Data sources | Cochrane Library | | |
| Search strategy | #1 | 287,712 | MeSH descriptor: [Aged] in all MeSH products |
|  | #2 | 75,666 | ("older adult" OR "elderly" OR "aging population" OR "senior") |
|  | #3 | 341,341 | #1 OR #2 |
|  | #4 | 122,617 | ("Special Health Care Need*" OR "medically complex" OR "chronic disease" OR "disability" OR "long-term care" OR "home care" OR "family caregiver*" OR "chronic disease*" OR "disab*" OR "cognitive impairment" OR "medical depend*" OR "caregiver") |
|  | #5 | 166 | MeSH descriptor: [Natural Disasters] explode all trees |
|  | #6 | 94,333 | ("emergency" OR "hazard*" OR "flood" OR "earthquake" OR "pandemic") |
|  | #7 | 94,411 | #5 OR #6 |
|  | #8 | 36,551 | ("information need*" OR "risk communication" OR "warning information" OR "emergency communication" OR "communication" OR "health information" OR "information behavior" OR "information seeking" OR "information access" OR "health communication") |
|  | #9 | 86 | #3 AND #4 AND #7 AND #8 |

| Item | Content | | |
| --- | --- | --- | --- |
| Data sources | Ebsco | | |
| Search strategy | #1 | 2,044,286 | ("Aged" OR "older adult*" OR "elderly" OR "aging population" OR "senior" ) |
|  | #2 | 1,138,571 | ("Special Health Care Need*" OR "medically complex" OR "chronic disease" OR "disability" OR "long-term care" OR "home care" OR "family caregiver*" OR "chronic disease*" OR "disab*" OR "cognitive impairment" OR "medical depend*" OR "caregiver") |
|  | #3 | 1,013,073 | ("Disaster*" OR "emergency" OR "hazard*" OR "flood" OR "earthquake" OR "pandemic") |
|  | #4 | 1,182,671 | ("information need*" OR "risk communication" OR "warning information" OR "emergency communication" OR "communication" OR "health information" OR "information behavior" OR "information seeking" OR "information access" OR "health communication") |
|  | #5 | 25 | #1 AND #2 AND #3 AND #4 |

| Item | Content | | |
| --- | --- | --- | --- |
| Data sources | CNKI | | |
| Search strategy | #1 | 388,300 | SU= (老年人 + 老龄人群 + 高龄者 + 老人) |
|  | #2 | 92,073 | SU= (特殊医疗需求 + 慢性病 + 残疾 + 长期照护 + 居家护理 + 家庭照护 + 慢病 + 失能 + 医疗依赖 + 慢性病) |
|  | #3 | 553,180 | SU= (灾害 + 突发事件 + 应急 + 灾难 + 自然灾害 + 公共卫生事件) |
|  | #4 | 56,891 | SU= (信息需求 + 风险沟通 + 预警信息 + 应急信息 + 信息行为 + 信息获取 + 信息沟通 + 信息支持 + 知识需求) |
|  | #5 | 7 | #1 AND #2 AND #3 AND #4 |

| Item | Content | | |
| --- | --- | --- | --- |
| Data sources | 万方 | | |
| Search strategy | #1 | 668,540 | (老年人 OR 老龄人群 OR 高龄者 OR 老人) |
|  | #2 | 371,142 | (特殊医疗需求 OR 慢性病 OR 残疾 OR 长期照护 OR 居家护理 OR 家庭照护 OR 慢病 OR 失能 OR 医疗依赖 OR 慢性病) |
|  | #3 | 149,201 | (灾害 OR 突发事件 OR 应急 OR 灾难 OR 自然灾害 OR 公共卫生事件) |
|  | #4 | 14,731 | (信息需求 OR 风险沟通 OR 预警信息 OR 应急信息 OR 信息行为 OR 信息获取 OR 信息沟通 OR 信息支持 OR 知识需求) |
|  | #5 | 15 | #1 AND #2 AND #3 AND #4 |

| Item | Content | | |
| --- | --- | --- | --- |
| Data sources | CBM | | |
| Search strategy | #1 | 34,013 | (老年人 OR 老龄人群 OR 高龄者 OR 老人) |
|  | #2 | 92,073 | (特殊医疗需求 + 慢性病 + 残疾 + 长期照护 + 居家护理 + 家庭照护 + 慢病 + 失能 + 医疗依赖 + 慢性病) |
|  | #3 | 553,180 | SU= (灾害 + 突发事件 + 应急 + 灾难 + 自然灾害 + 公共卫生事件) |
|  | #4 | 56,891 | SU= (信息需求 + 风险沟通 + 预警信息 + 应急信息 + 信息行为 + 信息获取 + 信息沟通 + 信息支持 + 知识需求) |
|  | #5 | 9 | #1 AND #2 AND #3 AND #4 |
